# Spatially modulated visuomotor efficiency in FPS players: eye-tracking evidence from a Go/No-Go target acquisition task

**DOI:** 10.1186/s41235-026-00738-6

**Published:** 2026-05-28

**Authors:** Liu Yang, Yuru Huang, Peitao Li, Hongjie Tang, Xinhong Jin

**Affiliations:** 1 School of Psychology, Shanghai University of Sport, Shanghai, China; 2 Center for Exercise and Brain Science, Shanghai University of Sport, Shanghai, China; 3 Key Laboratory of Motor Cognitive Assessment and Regulation, Shanghai, China

**Keywords:** First-person shooter games, Spatial attention, Eye movement, Visuomotor efficiency, Go/No-Go task

## Abstract

First-person shooter (FPS) games demand rapid visual processing, spatial awareness, and response inhibition, yet the cognitive correlates of FPS gameplay remain under debate. This study examined whether FPS gaming experience is associated with inhibitory control indices and visuomotor efficiency using a gamified Go/No-Go target acquisition task with FPS-relevant spatial demands. Behavioral and eye movement indices were compared between 29 experienced FPS players (16 males; mean age = 21.38 ± 2.11 years) and 32 non-FPS participants (17 males; mean age = 21.00 ± 2.11 years). Results showed that FPS players demonstrated reliably faster execution times, while accuracy on Go trials did not differ between groups. For No-Go trials, overall commission error rates did not show a significant group effect, but were systematically modulated by spatial context (quadrant and its interaction with target distance). Eye-tracking results further revealed more efficient oculomotor dynamics in FPS players, including shorter saccade latencies and durations, with group differences most pronounced in specific spatial locations (notably Quadrant 2). Together, these findings suggest that FPS gaming experience is associated with faster visuomotor responding and distinct gaze dynamics under spatially distributed demands, while inhibitory accuracy appears more strongly shaped by spatial constraints than by experience level.

## Significance statement

Competitive first-person shooter (FPS) games are fast-paced and visually complex, requiring players to quickly detect targets, make split-second decisions, and avoid incorrect actions. These demands reflect real-world challenges in environments such as driving, emergency response, or military operations. Our study investigates whether experience with FPS games leads to measurable improvements in cognitive control and visual attention. Using a gamified Go/No-Go target acquisition task that incorporates FPS-relevant spatial demands, we compared FPS-experienced participants and non-FPS participants on their ability to respond quickly and accurately and to withhold responses when required. We also tracked eye movements to examine how attention is allocated across space during target acquisition. FPS-experienced participants showed faster responses and more efficient gaze dynamics, particularly under spatially demanding conditions, whereas inhibitory accuracy was influenced primarily by target distance and quadrant location rather than a uniform group advantage. These findings suggest that certain forms of interactive digital experience may be linked to measurable differences in visuomotor efficiency and spatial attention, with potential relevance beyond gaming. By connecting everyday digital behavior to foundational cognitive processes, our work helps explain how people adapt to complex visuomotor environments and informs the design of ecologically valid assessments for education, training, and interface design.

## Introduction

With the rapid advancement of digital technology, video games have become a dominant form of entertainment, and esports have grown into a global phenomenon (Bányai et al., 2019). Among various video games, first-person shooter (FPS) games stand out for their fast-paced and high-intensity demands. Players must process complex visual information, make split-second decisions, and adapt flexibly to dynamic environments. Research suggests that engagement with such cognitively demanding games may enhance attentional control, executive function, and decision-making skills (Griffiths et al., 2004; Zhong, 2011). As video games continue to shape modern life, their potential influence to cognitive performance, especially through FPS gameplay, has become an important area of investigation.

FPS games require players to rapidly detect and engage dynamic targets while constantly shifting between focused attention on specific threats and broader situational awareness. Successful play relies on fast decision making, the suppression of prepotent responses when appropriate, and continuous adjustment of actions to changing scenarios. Consistent with these demands, experienced FPS players often outperform non-players on FPS-relevant visuomotor components, including target acquisition and visual search, which has been linked to more efficient eye movement patterns and enhanced visuospatial attention (Campbell et al., 2018; Kowal et al., 2018; Yang et al., 2025). FPS gameplay has also been associated with faster execution times, improved attentional control, and more efficient information processing (Anguera et al., 2013; Hutchinson et al., 2016). However, findings regarding inhibitory control remain mixed. Some studies report that FPS players show improvements in working memory without corresponding gains in action inhibition (Colzato et al., 2013), whereas others suggest that extensive action video game engagement may be related to heightened impulsivity or reduced cognitive control in certain samples (Deleuze et al., 2017; Metcalf & Pammer, 2014). Overall, the literature suggests reliable advantages in top-down attention and visuomotor coordination among action video game players (Li et al., 2016; Wu & Spence, 2013), but it remains unclear whether FPS gaming experience is consistently associated with inhibitory accuracy, especially under spatially demanding conditions (Cardoso-Leite et al., 2016; Dobrowolski et al., 2015).

In FPS gameplay, players are frequently confronted with the need to rapidly distinguish between enemies and teammates under conditions of high visual uncertainty. A critical moment occurs when a target appears suddenly: If it is an enemy, an immediate attack is required; if it is a teammate, the player must inhibit the shooting response. Failing to do so, committing so-called friendly fire, can negatively affect game outcomes, highlighting the importance of fast and accurate response of friendly fire. To capture these demands in an experimentally controlled manner, we employed a modified Go/No-Go paradigm embedded in a mouse-based target acquisition (point-and-click shooting) task adapted from prior work (Yang et al., 2025). In this task, participants were instructed to click red targets (Go) and to withhold responding to blue targets (No-Go). Importantly, target location varied systematically as a function of distance from fixation and angular position, allowing us to examine how spatial context modulates behavioral performance and eye movement dynamics. This design enables a fine-grained test to investigate the association between FPS gaming experience with response execution, inhibitory control indices, and visuomotor efficiency in spatially distributed contexts that mirror the demands of competitive gameplay (Castel et al., 2005; Hershler & Hochstein, 2009).

Eye-tracking technology has emerged as a powerful tool for objectively assessing visual search strategies and attentional control, particularly through metrics such as fixation and saccadic patterns (Brams et al., 2019). Compared to self-report or behavioral measures, eye-tracking provides finer data on how individuals allocate visual attention during dynamic tasks. The aiming advantages of experienced FPS players stem from their more effective eye movement patterns (Yang et al., 2025). Prior research has shown that experts typically demonstrate more efficient visual search strategies, including shorter saccades, fewer fixations, and more direct visual orientation in complex visual scenarios (Brams et al., 2019; Eckstein, 2011; Holm et al., 2021; Papesh et al., 2021). However, most existing studies have focused on general visual search without systematically considering how spatial variables (such as target location and distance) modulate inhibitory control and eye-tracking performance. Moreover, experimental comparisons between FPS players and non-gamers often lack ecological validity or consistent task structure (Azizi et al., 2017; Loh et al., 2015; Marre et al., 2021).

To address these limitations, the present study integrates eye-tracking with a gamified mouse-based target acquisition task that embeds Go/No-Go demands, enabling a fine-grained examination of how FPS gaming experience relates to visuomotor efficiency and inhibitory control indices across spatially distributed conditions. Specifically, we employed a modified Go/No-Go paradigm in which participants made rapid mouse click responses to color-coded targets presented across multiple target distances from fixation and quadrants, allowing us to quantify both behavioral performance (execution time, Go accuracy, and No-Go commission errors) and gaze dynamics (e.g., saccade latency and duration) within the same framework. Based on the visuomotor demands of FPS play, we expected experience-related differences to be most evident in response execution speed and oculomotor efficiency, while predictions regarding overall No-Go commission errors remained exploratory given mixed prior evidence. Instead, we anticipated that target distance and quadrant location would systematically modulate both behavioral performance and eye movements, and we tested whether any experience-related differences were amplified under specific spatial demands. More broadly, by combining eye-tracking with a spatially structured Go/No-Go target acquisition task, the present study provides an ecologically grounded and process-level approach to examining how spatial attention and response control operate in FPS-relevant visuomotor settings.

## Methods

### Participants

Before the experiment, participants completed a screening questionnaire assessing their gaming habits, including preferred game genres, cumulative FPS gaming experience, and average weekly playtime. Experienced FPS players were defined as individuals with at least two years of regular FPS gameplay experience who had played FPS games at least once per week during the past six months. Non-FPS participants reported no prior FPS gaming experience and responded “No” to the question “Do you play FPS games?”.

The final sample size was determined based on participant availability and recruitment feasibility during the study period, particularly given the requirement to recruit individuals with specific FPS gaming experience criteria while maintaining comparable group characteristics across groups. To ensure robustness, a total of 61 volunteers (33 males, 28 females; mean age = 21.16 ± 2.03 years) were recruited from campus through public announcements. Among them, 29 were experienced FPS players (16 males; mean age = 21.38 ± 2.11 years), with an average of 3.83 ± 1.31 years of FPS gaming experience, playing approximately 2.78 ± 0.64 times per week for 7.59 ± 3.34 h weekly. These players used either computers (n = 14) or mobile phones (n = 15). The remaining 32 participants (17 males; mean age = 21.00 ± 2.11 years) had no prior FPS gaming experience. No significant age difference was found between the two groups, *t*(59) = 0.661, *p* = 0.545. The detailed gaming habits of FPS players are presented in Table 1. All participants were right-handed and had no known neurological impairments. Participants provided written informed consent and were assured anonymity. The study was approved by the Scientific Research Ethics Committee of Shanghai University of Sport (No. 102772022RT080) and conducted in accordance with the Declaration of Helsinki. Monetary compensation was provided upon completion.

**Table 1:** Descriptive statistics for FPS gaming experience

Item	Mean	SD
How many years played	3.83	3.45
How many times a week	2.78	1.69
How many hours each time	2.00	1.10
How many hours a week	7.59	8.78

The means (± standard deviations) for years of FPS gameplay, weekly frequency, session duration, and total weekly hours are presented for the FPS group

### Apparatus and stimuli

Eye movements were recorded using the EyeLink 1000 Plus (SR Research Ltd, Canada) at a sampling rate of 1000 Hz during the aiming task. A nine-point calibration was performed at the start of the experiment and after each break. To ensure successful calibration, participants were required to maintain fixation within a 1.5° × 1.5° window around the fixation point for at least 200 ms. If calibration failed three times, it was repeated until successful. Data collection commenced upon successful calibration. The stimuli were presented on a 24-inch monitor (1024 × 768 resolution, 60 Hz refresh rate) positioned 57 cm from the participants. A chinrest was used to minimize head movements and ensure stability during the task.

### Procedure

The experimental task was developed in MATLAB and PsychToolbox (version 3.0.19). The paradigm was designed as a gamified, mouse-based target acquisition Go/No-Go task that preserves key FPS-relevant demands (rapid target detection/selection and response inhibition) while maintaining experimental control. At the beginning of the experiment, participants were provided with written instructions and performed a nine-point eye-tracking calibration. They then completed 10 practice trials, followed by an additional calibration to ensure data accuracy before the formal task commenced.

Each trial began with a central fixation cross presented for 500-700 ms, ensuring that participants initially fixated on the center of the screen. Following fixation offset, a target stimulus appeared at one of eight angular positions (0°, 45°, 90°, 135°, 180°, 225°, 270°, and 315°, clockwise) and at one of three target distances from the screen center (near, medium, far). Target eccentricities were 150 pixels (7.14 cm; approximately 7.2° of visual angle), 300 pixels (14.29 cm; approximately 14.3° of visual angle), and 450 pixels (21.43 cm; approximately 21.3° of visual angle), based on a viewing distance of 57 cm.

**Fig. 1:**
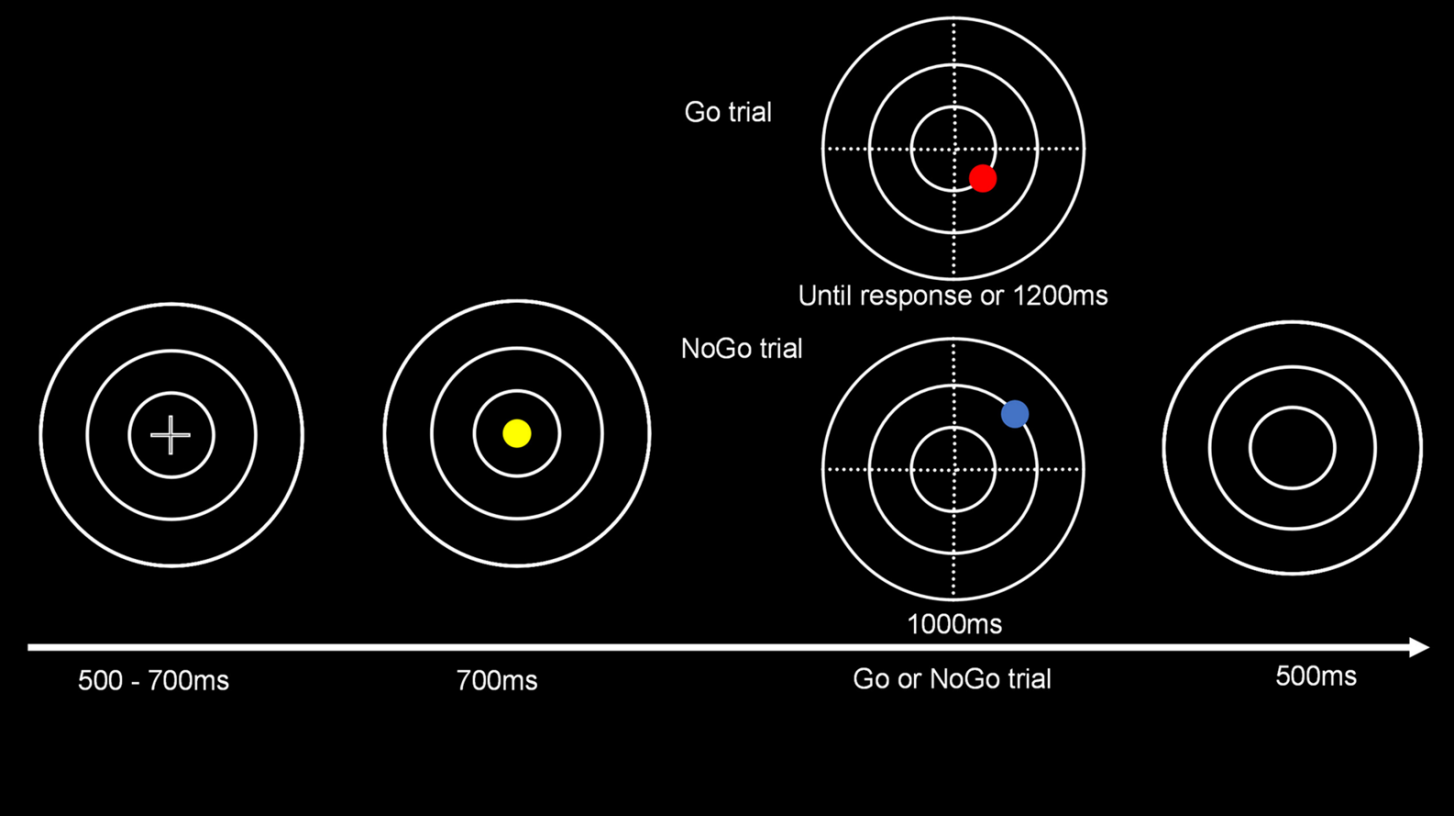
Structure and timeline of the gamified Go/No-Go target acquisition task. Each trial began with a central fixation cross presented for 500–700 ms, followed by a centrally located yellow dot displayed for 700 ms that participants were required to click with the mouse to initiate the trial and standardize the starting position. Subsequently, a target stimulus appeared at one of three distances and one of eight angular locations (0°, 45°, 90°, 135°, 180°, 225°, 270°, 315°). On Go trials (red target), participants were instructed to respond as quickly and accurately as possible by clicking the target; responses were recorded until the click or a 1200-ms deadline, with failures classified as misses. On No-Go trials (blue target), participants were instructed to withhold responding; any mouse click during the 1000-ms presentation window was recorded as a commission error

On Go trials (red target), participants were instructed to click the target with the mouse as quickly and accurately as possible. Execution time (ms) was defined as the interval between target onset and the mouse click; responses slower than 1200 ms were classified as misses. On No-Go trials (blue target), participants were instructed to withhold responding; any mouse click during the No-Go window was coded as a commission error. Eye movement measures were recorded throughout target processing in both trial types. The Go/No-Go trial ratio was 2:1 to maintain task engagement (see Fig. 1 for more details). Participants were instructed to fixate on the central fixation point at the beginning of each trial. After target onset, eye movements were not constrained, allowing natural gaze shifts during target acquisition. Because targets appeared unpredictably across target distances and spatial locations, successful performance typically involved saccades toward the target position, enabling assessment of oculomotor dynamics under spatially distributed visuomotor demands.

### Data processing and analysis

All data analyses were conducted using R (version 4.4.0), with the eye-linker package (version 0.2.1) used to import and process plain-text ASC data files from the eye tracker. Accuracy rate was defined as the proportion of correctly executed responses within the time limit in Go trials. Execution time, measured as the average reaction time in Go trials, was calculated from target onset to the participant’s successful response. Error rate in No-Go trials was defined as the proportion of incorrect responses, representing the frequency of mouse hitting when no response was required. These behavioral measures collectively provide insight into participants’ response execution and inhibitory control.

Additionally, a series of linear eye movement metrics were recorded and analyzed to examine differences in visuomotor processing during task execution. Eye movement data were automatically encoded using eye-tracking software, with fixation and saccade definitions based on established thresholds. Specifically, the analyzed metrics included total fixation count, total saccade count, average fixation duration, average saccade duration, saccade latency, and average saccade amplitude. Fixation was defined as a period during which gaze remained within 1.0° of visual angle for at least 50 ms, while a saccade was classified as an eye movement with a velocity exceeding 30°/s, an acceleration greater than 8000°/s^2^, and a minimum movement threshold of 0.15°. Saccade latency was defined as the time interval from target onset to the initiation of the first saccade. All fixation and saccade thresholds followed the default settings of the EyeLink 1000 Plus (SR Research, Canada).

To examine group differences and interactions between task variables, a three-factor repeated-measures analysis of variance (ANOVA) was conducted to analyze the main effects of group FPS gaming experience, target distance, and target quadrant and their interactions. In the ANOVA, when Mauchly’s test of sphericity showed heterogeneity of covariance, the more conservative Greenhouse-Geisser test was performed. Statistical significance was set at *p* < 0.05, with Bonferroni corrections applied for significant ANOVA factors. Analyses were conducted using SPSS 27.0.

## Results

### Behavioral results

*Accuracy.* The three-factor interaction effect of group × target distance × target quadrant was not significant, *F* (6,354) = 0.697, *p* = 0.652. The interaction effects of group × target quadrant, *F* (3,177) = 0.670, *p* = 0.572, group × target distance, *F*(2,118) = 0.467, *p* = 0.628, target distance × target quadrant, *F*(6,354) = 0.775, *p* = 0.590, were not significant. The main effect of target distance was significant, *F*(2,118) = 29.714, *p* < 0.001, *η*_*p*_^*2*^ = 0.336. The post hoc test of distance showed that the accuracy of near distance was higher than that of medium distance (*p* = 0.009), higher than that of far distance (*p* < 0.001), and the accuracy of medium distance was also significantly higher than that of far distance (*p* < 0.001). And the main effect of target quadrant was significant, *F* (3,177) = 3.836, *p* = 0.011, *η*_*p*_^*2*^ = 0.061. The post hoc test results showed that the accuracy of the quadrant 2 was higher than that of the quadrant 3 (*p* = 0.001), the quadrant 4 was higher than that of the quadrant 3 (*p* = 0.006), and there was no significant difference in other quadrants.

*Execution time.* The interaction of group × target distance × target quadrant was not significant, *F*(6, 354) = 0.224, *p* = 0.962. The interaction effects of group × target quadrant, *F*(3,177) = 0.068, p = 0.977, group × target distance, *F*(2, 118) = 0.286, *p* = 0.751, target distance × target quadrant, *F*(6,354) = 1.592, *p* = 0.148, were not significant. The main effect of group was significant, *F*(1,59) = 5.108, *p* = 0.028, *η*_*p*_^*2*^ = 0.080. The execution time of the FPS group was significantly faster than that of the non-FPS group. There were also significant main effects of target distance, *F*(2,118) = 298.077, *p* < 0.001, *η*_*p*_^*2*^ = 0.835; and target quadrant, *F*(3,177) = 5.255, *p* = 0.002, *η*_*p*_^*2*^ = 0.082, on execution times. In the post hoc test of the quadrant, the execution time of the quadrant 2 was faster than that of the quadrant 1 (*p* = 0.008) and quadrant 3 (*p* = 0.001), and the quadrant 4 was faster than that of the quadrant 3 (*p* = 0.013). Moreover, the execution time at near distance was faster than that at medium distance (*p* < 0.001) and far distance (*p* < 0.001), and the execution time at medium distance was faster than that at the far distance (*p* < 0.001).

*Error rate.* The three-factor interaction effect of group × target distance × target quadrant was significant for No-Go error rates, *F*(6,354) = 2.188, *p* = 0.044, *η*_*p*_^*2*^ = 0.036. Comparisons indicated that within the FPS group, No-Go error rates in quadrant 1 were lower at medium distances than at far distances (*p* = 0.001), in quadrant 2 were lower at near distances than at medium distances (*p* = 0.024). No other distance-related differences were observed within the FPS group, and no significant effects emerged for No-Go error rates in the non-FPS group. The interaction effects of group × target quadrant, *F*(3,177) = 0.163, *p* = 0.921, group × target distance, *F*(2,118) = 0.176, *p* = 0.839, were not significant. However, there was a significant interaction effect between target distance × target quadrant, *F*(6,354) = 3.442, *p* = 0.003, *η*_*p*_^*2*^ = 0.055. The post hoc test showed no significant difference between near and far distances, suggesting that the error rate was not affected by quadrants in the case of near and far distances. However, in the medium distance, there was a significant difference between quadrant 1 and quadrant 2 ( *p* = 0.001), which showed that the error rate in quadrant 2 was higher than that in quadrant 1, and there was a significant difference between quadrant 2 and quadrant 3 (*p* = 0.016), which showed that quadrant 2 was higher than quadrant 3, indicating that in the case of medium distance, the stimulus in quadrant 2 was more likely to be misjudged.

**Fig. 2:**
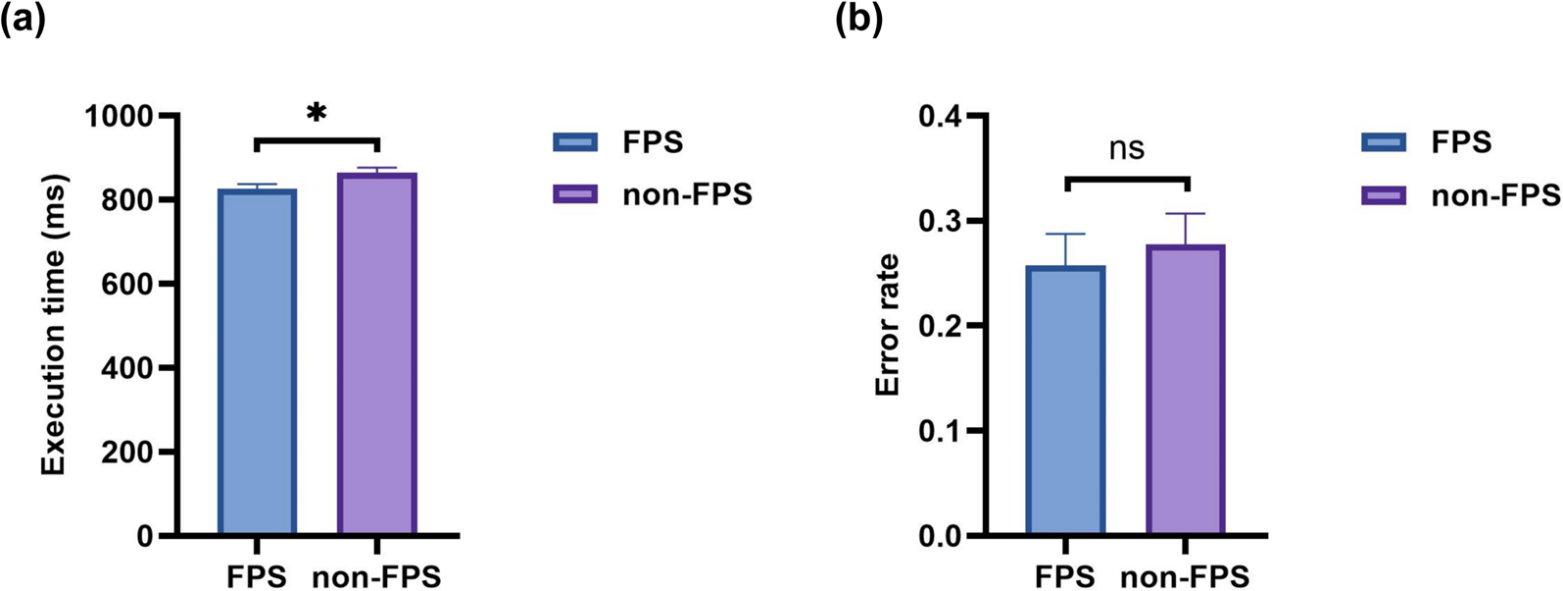
Behavioral performance in the Go/No-Go target acquisition task for FPS and non-FPS participants. Bar plots show (**a**) mean Go trial execution time (ms) and (**b**) mean No-Go commission error rate (proportion of mouse clicks on No-Go trials). Execution times are reported in milliseconds and are expected to be longer than those in standard centrally presented keypress Go/No-Go paradigms because the present task requires spatial orienting and mouse-based target acquisition to targets presented at varying distances and locations. Error bars represent standard errors of the mean (± SEM). *Note:* *p < .05; **p < .01; ***p < .001

Moreover, the main effect of group was not significant, *F*(1,59) = 0.228, *p* = 0.635. There was a significant main effect of target quadrant, *F*(3,177) = 4.170, *p* = 0.007, *η*_*p*_^*2*^ = 0.066. Post hoc comparisons showed that error rates were higher in quadrant 2 than in quadrant 1 (*p* = 0.014) and also higher than in quadrant 3 (*p* = 0.022). Error rates were also higher in quadrant 4 than in quadrant 1 (*p* = 0.028) and quadrant 3 (*p* = 0.006). The main effect of target distance was not significant, *F*(2,118) = 1.958, *p* = 0.146 (Fig. 2).

### Eye movement

**Fig. 3:**
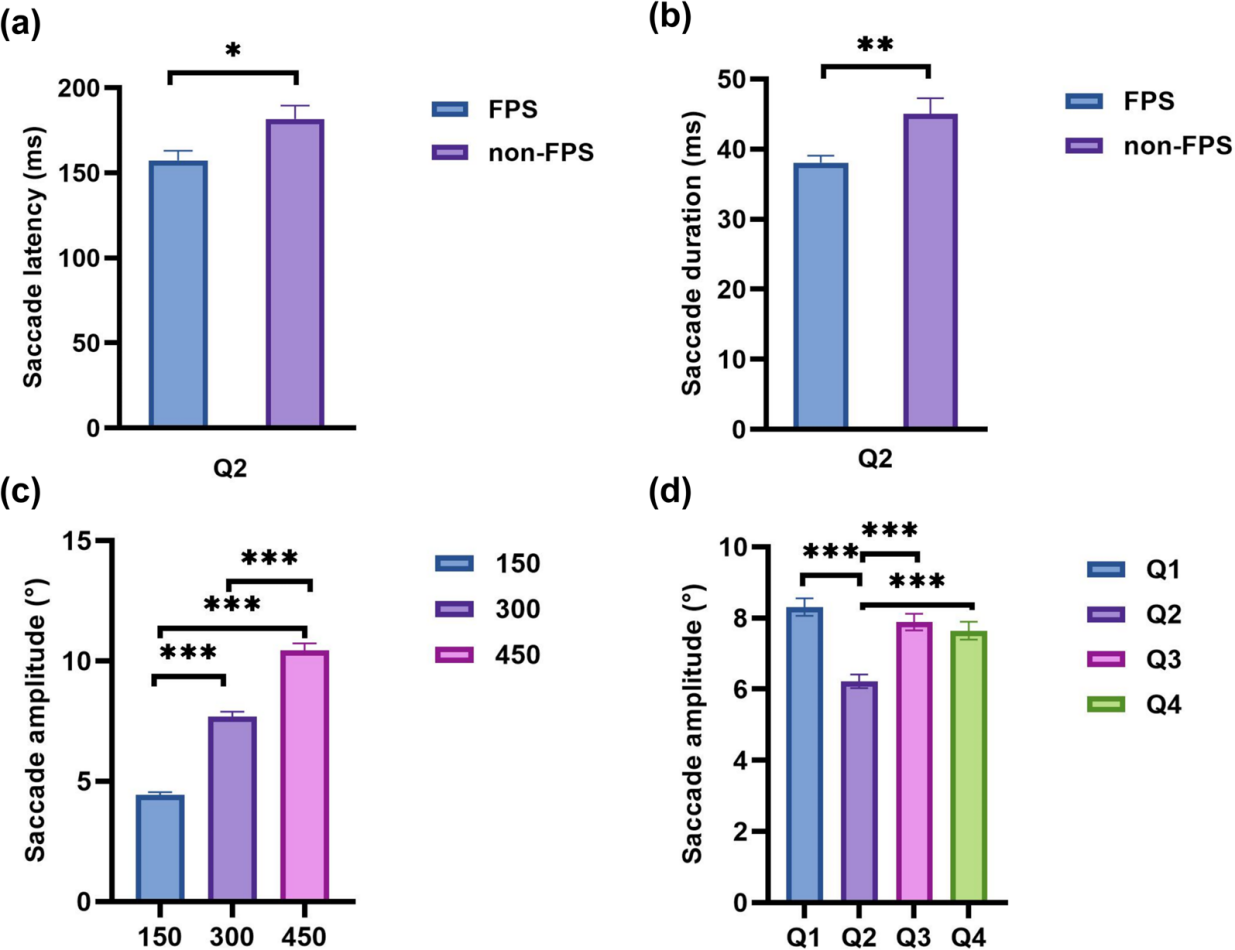
Eye movement measures in the Go/No-Go target acquisition task. (**a**) Saccade latency in quadrant 2 for FPS and non-FPS players; (**b**) saccade duration in quadrant 2 for FPS and non-FPS players; (**c**) saccade amplitude across target distances (near, medium, far); (**d**) saccade amplitude across spatial quadrants (Q1–Q4). All data are presented as group means ± standard error of the mean (SEM). Q1–Q4 refer to the four visual quadrants in the experimental display. *Note:* *p < .05; **p < .01; ***p < .001

Regarding the eye movement data, we extracted the data for both Go and No-go conditions. However, due to the limited number of trials and the high rate of missing values under the No-go condition, we only conducted statistical processing of the eye movement indicators for the Go trials (Fig. 3).

*Saccade amplitude*. The interaction of group × target distance × target quadrant was not significant, *F*(4.124,243.324) = 0.868, *p* = 0.487. The interaction between group and target quadrant was significant, *F*(3,177) = 3.367, *p* = 0.020, *η*_*p*_^*2*^ = 0.054; however, post hoc comparisons did not yield any significant pairwise differences. The target distance × target quadrant was significant, *F*(4.124,243.324) = 6.270, *p* < 0.001, *η*_*p*_^*2*^ = 0.096. Post hoc test found that in the case of near range, there were significant differences between quadrant 1 and quadrant 2 (*p* < 0.001), quadrant 1 and quadrant 4 (*p* = 0.009), quadrant 2 and quadrant 3 (*p* < 0.001), quadrant 2 and quadrant 4 (*p* < 0.001). Similar results were obtained at medium distances, with significant differences between the quadrant 1 and quadrant 2 (*p* < 0.001), quadrant 1 and quadrant 4 (*p* = 0.001), quadrant 2 and quadrant 3 (*p* < 0.001), quadrant 2 and quadrant 4 (*p* < 0.001). Under the condition of far distance, there were significant differences between quadrant 1 and quadrant 2 (*p* < 0.001), quadrant 2 and quadrant 3 (*p* < 0.001), quadrant 2 and quadrant 4 (*p* < 0.001), but there was no significant difference between quadrant 1 and quadrant 4. It showed that regardless of the distance, the saccade amplitude in the quadrant 1 was greater than that in the quadrant 2, and the quadrant 2 was lower than the quadrant 3 and quadrant 4. However, the saccade amplitude in the quadrant 1 was significantly larger than that in the quadrant 4 in the near and medium distance and did not show similar response in the far distance. The interaction effects of group and target distance, *F*(3.609,501.737) = 0.424, *p* = 0.584, were not significant.

In addition, the main effect of group was not significant, *F*(1,59) = 0.028, *p* = 0.868. There was a significant main effect of target quadrant on saccade amplitude, *F*(3,177) = 38.563, *p* < 0.001, *η*_*p*_^*2*^ = 0.395. Post hoc tests showed that the saccade amplitude in the quadrant 2 was lower than that in the quadrant 1 (*p* < 0.001), quadrant 3 (*p* < 0.001), and quadrant 4 (*p* < 0.001). The main effect of target distance was also significant, *F*(1.400,82.580) = 515.272, *p* < 0.001, *η*_*p*_^*2*^ = 0.897. The post hoc test results showed that there were significant differences between the near, medium, and far distances (*p* < 0.001); the near distance was significantly less than the medium and far distance; the medium distance was significantly less than the far distance.

*Saccade duration.* The interaction of group × target distance × target quadrant was not significant, *F*(4.224,249.233) = 1.400, *p* = 0.214. The interaction effects of target distance × target quadrant, *F*(4.224,249.233) = 1.102, *p* = 0.360, group × target distance, *F*(1.818,107.242) = 0.166, *p* = 0.847, were not significant. But, group × target quadrant was significant, *F*(3,177) = 3.666, *p* = 0.013, *η*_*p*_^*2*^ = 0.059. The post hoc results showed that there was a significant difference between the FPS group and the non-FPS group only in the 2 quadrant (*p* = 0.007) and showed that participants with FPS gaming experience had a shorter duration than those without experience.

And, the main effect of group was not significant, *F*(1,59) = 0.707, *p* = 0.404. There was a significant main effect of target quadrant on saccade duration, *F*(3,177) = 3.804, *p* = 0.011, *η*_*p*_^*2*^ = 0.061. The main effect of target distance was also significant, *F*(1.818,107.242) = 117.001, *p* < 0.001, *η*_*p*_^*2*^ = 0.665. The post hoc test showed that the saccade duration in the quadrant 2 was significantly shorter than that in the quadrant 1 (*p* = 0.023) and quadrant 4 (*p* = 0. 003), and the short distance, medium distance, and far distance were significantly different from each other (*ps* < 0.001).

*Saccade latency.* The interaction of group × target distance × target quadrant was not significant, *F*(6,354) = 0.469, *p* = 0.806. The interaction effects of target distance × target quadrant, *F*(6,354) = 0.571, *p* = 0.753, group × target distance, *F*(2,118) = 0.319, *p* = 0.728, were not significant. However, group × target quadrant was significant, *F*(3,177) = 3.963, *p* = 0.009, *η*_*p*_^*2*^ = 0.063. The post hoc results showed significant differences were found only in the quadrant 2 between FPS group and the non-FPS group (*p* = 0.016), and participants with FPS gaming experience had less latency than those without experience.

And, the main effect of group was not significant, *F*(1,59) = 1.391, *p* = 0.243. There was a significant main effect of target quadrant, *F*(3,177) = 27.860, *p* < 0.001, *η*_*p*_^*2*^ = 0.321. Post hoc test showed that the latency of quadrant 1 was higher than quadrant 2 (*p* < 0.001) and quadrant 3 (*p* < 0.001), shorter than quadrant 4 (*p* = 0.006), quadrant 2 (*p* < 0.001), and quadrant 3 (*p* < 0.001), shorter than quadrant 4. The main effect of target distance was not significant, *F*(2,118) = 1.966, *p* = 0.145.

## Discussion

The present study examined how FPS gaming experience is associated with behavioral performance and eye movement dynamics in a spatially structured Go/No-Go target acquisition task. Overall, experienced FPS players responded faster on Go trials, and eye-tracking measures indicated more efficient oculomotor dynamics during target acquisition (e.g., shorter saccade latencies and durations). In contrast, overall No-Go commission error rates did not show a reliable main effect of group; instead, inhibitory performance was primarily shaped by spatial context. Target distance and quadrant systematically modulated execution times, and No-Go errors varied as a function of quadrant and its interaction with target distance, with selective distance-related differences emerging within the FPS group. Together, these findings suggest that FPS experience is associated with faster visuomotor responding and distinct gaze strategies under spatially distributed demands, while inhibitory accuracy appears more sensitive to spatial constraints than to group membership.

Accuracy and execution time in response to the Go signal serve as key indicators of task performance under response execution demands. Go accuracy did not significantly differ between FPS players and non-FPS participants; however, it was systematically influenced by spatial factors, with performance declining as target distance increased and varying across quadrants. This suggests that spatial constraints influence perceptual and motor processing in both groups. In contrast, execution time exhibited a group difference, with FPS players demonstrating significantly faster execution times across all conditions. This aligns with previous research showing that FPS players respond more quickly than non-players in both feature and conjunction search tasks, despite similar accuracy levels (Colzato et al., 2013; Wu & Spence, 2013; Yang et al., 2025). Beyond group effects, execution times were strongly modulated by target distance and quadrant location, indicating that spatial context imposes substantial processing costs even in Go trials. Faster responses in FPS players across target distances and quadrants may reflect more efficient stimulus-response translation and visuomotor planning in FPS-relevant target selection (Castel et al., 2005). This pattern may reflect the task demands characteristic of FPS gameplay, which require rapid decision-making and continuous sensorimotor coordination and are known to engage neural systems related to attentional control and action planning (Lin et al., 2025). Importantly, the current paradigm differs from standard centrally presented keypress Go/No-Go tasks: Responses required mouse-based target acquisition (i.e., clicking spatially distributed targets), such that execution times reflect not only response selection but also visuospatial orienting and goal-directed pointing. Accordingly, the absolute RT values should be interpreted in relation to these additional spatial and motor demands rather than benchmarks from simple keypress Go/No-Go paradigms.

No-Go performance was quantified as commission errors (mouse clicks on No-Go trials) within the response window. In the present task, inhibitory accuracy did not show a reliable overall difference between FPS players and non-FPS participants; instead, No-Go error rates were systematically shaped by spatial context. Specifically, error rates varied across target quadrants and exhibited a quadrant-by-target-distance interaction, with quadrant differences emerging most clearly at the medium target distance. Moreover, within the FPS group, distance-related changes in error rates occurred in specific quadrants, whereas no comparable effects were observed within the non-FPS group. Together, these patterns indicate that inhibitory performance in this paradigm is more strongly constrained by spatial demands than by gaming experience per se. This finding helps reconcile inconsistencies in the literature on action video games and inhibitory control. Some prior work, particularly studies focusing on excessive or problematic gaming, has linked heavy video game use to poorer inhibitory performance (e.g., higher commission errors) alongside faster responding on Go trials (Fathi et al., 2024). In contrast, studies using conventional, centrally presented keypress Go/No-Go paradigms sometimes report small or inconsistent gamer advantages and in many cases no reliable improvements in inhibition. One plausible contributor to these mixed results is task difficulty and response modality. Our paradigm required mouse-based target acquisition to spatially distributed targets, which increases visuospatial uncertainty and imposes additional orienting and motor-planning demands relative to standard Go/No-Go tasks. Under such conditions, inhibitory errors may be driven less by a general “inhibitory capacity” and more by spatially specific perceptual constraints and speed-accuracy tradeoffs. The spatial pattern of No-Go errors is also consistent with known heterogeneity in visual resolution across the visual field. Visual acuity is highest near the fovea/macula and decreases with increasing retinal eccentricity, which can impair target discrimination and response selection for more peripheral stimuli (Bringmann et al., 2018; Kowler, 2011). Although we did not directly manipulate fixation strategy, the observed quadrant and distance effects suggest that spatial visibility constraints contribute meaningfully to commission errors in the present task, especially at intermediate target distances where perceptual uncertainty may be greatest.

Our eye-tracking results suggest that while FPS and non-FPS players exhibit differences in eye movement patterns, these differences are modulated by spatial factors rather than being directly significant. Saccade amplitude, which reflects the angle of movement during a saccade, was influenced by both quadrant and distance. Regardless of group membership, saccade amplitude was smallest at short distances and largest at far distances, following the expected relationship between target proximity and required eye movement. Additionally, within the same distance, saccade amplitude varied across quadrants, likely due to differences in spatial dominance. These findings align with previous research showing that experienced FPS players demonstrate superior target localization efficiency, primarily due to their optimized visual search strategies (Li et al., 2009). However, in our study, while FPS players displayed differences in eye movement indicators, these differences did not reach significance, suggesting that quadrant- and distance-related effects may overshadow the impact of gaming experience (Li et al., 2022).

In contrast, saccade duration exhibited a direct group difference, modulated by quadrant effects. Prolonged saccade durations have been associated with increased cognitive load and greater involvement of top-down attentional processes, whereas shorter saccade durations are more often linked to faster oculomotor execution and bottom-up driven visual exploration (Devillez et al., 2020; Han et al., 2025; Kowler, 2011). In the present study, FPS players showed significantly shorter saccade durations in the second quadrant compared to the first and fourth quadrants, whereas no comparable quadrant-related modulation was observed in the non-FPS group. This pattern is consistent with differences in visual attention allocation and eye movement control between FPS players and non-FPS participants, suggesting that FPS gaming experience is associated with more efficient saccadic execution in specific visual fields under spatially demanding conditions (Boot et al., 2008; Chisholm & Kingstone, 2015; Green & Bavelier, 2006). These differences in saccadic behavior may underlie the distinct performance patterns observed in behavioral tasks.

Saccade latency, which measures the time required for visual processing and motor response preparation, also exhibited interactions between group membership and quadrant. Both groups demonstrated similar latency patterns, with the shortest latencies in quadrant 2 and the longest in quadrant 4. This suggests that while FPS players may have faster motor responses in certain conditions, their overall efficiency in visual information processing remains comparable to non-FPS players. Prior research indicates that variations in object processing efficiency across different visual field locations may influence visual attention strategies (Galenchik-Chan et al., 2023). While video game players (VGPs) and non-video game players (NVGPs) may initially respond similarly to salient stimuli, VGPs do not necessarily exhibit superior goal-directed strategies for target selection (Sharp et al., 2022). This aligns with our findings, where FPS gaming experience influenced certain eye movement parameters without always leading to significant performance differences (Heimler et al., 2014). Notably, FPS players have been found to exhibit slightly lower error rates in the antisaccade task, potentially due to more efficient motor control (Mack & Ilg, 2014), which may partially explain why their eye movement performance did not always diverge significantly from non-players. In general, eye movement metrics tend to be negatively correlated with task performance, as increased saccade counts and prolonged latencies often indicate higher cognitive load or less efficient search strategies (Brams et al., 2019). In FPS-like screen-based tasks, longer quiet eye durations have been linked to enhanced aiming accuracy (Dahl et al., 2021). Action video game players are known to adopt more effective search strategies in gaming-related tasks (Azizi et al., 2017). Our findings support the notion that FPS players demonstrate distinct eye movement patterns compared to non-gamers. However, these differences are influenced by the interplay between gaming experience and spatial positioning, rather than representing a uniform advantage across all conditions. This suggests that while FPS gaming may refine certain aspects of visual processing, its effects are context-dependent and modulated by spatial and task-related constraints.

The present findings provide a complementary perspective to prior studies that reported null or inconsistent differences in inhibitory control between FPS players and non-players (Colzato et al., 2013; Deleuze et al., 2017; Steenbergen et al., 2015). Notably, much of this work has relied on standard inhibition paradigms (e.g., stop-signal tasks) that are decontextualized from the perceptual-motor demands characteristic of FPS gameplay. Emerging evidence suggests that contextualizing or gamifying classical inhibition tasks can meaningfully alter task engagement and experiential factors. For example, Friehs et al. (2020) showed that a gamified stop-signal task increased intrinsic motivation and flow, even when behavioral stopping performance did not change, highlighting how “use-inspired” task designs may capture control processes under more ecologically relevant conditions.

An important question is whether the experience-related patterns observed here reflect near transfer to tasks that share surface and sensorimotor features with FPS gameplay, or far transfer to broader cognitive abilities. Because our paradigm incorporated FPS-relevant demands (spatially distributed targets, rapid mouse-based target acquisition, and response selection under Go/No-Go requirements), the present effects are most consistent with near transfer, particularly given that group differences were most robust in response execution speed and oculomotor dynamics under spatially demanding conditions, whereas overall No-Go commission error rates did not show a reliable main effect of group. In this paradigm, inhibitory accuracy may be constrained more by spatial visibility and motor demands than by generalized inhibitory capacity. At the same time, our goal was not simply to reproduce an in-game advantage, but to isolate component processes, such as spatial attention allocation, gaze orienting, and speed–accuracy regulation, within a controlled experimental framework. Broader generalizability beyond FPS-like tasks remains to be established, and future training and longitudinal studies using paradigms with reduced surface similarity will be necessary to evaluate far transfer.

This study has several limitations. First, the present study was cross-sectional and did not include a training or intervention component. Therefore, although we sought to match the two groups on key characteristics, the observed between-group differences should be interpreted as associative rather than causal. Future research should employ randomized controlled training designs or longitudinal follow-ups to determine whether FPS-style training produces measurable changes in oculomotor efficiency and to establish the temporal dynamics and dose-response relationship of any training effects. Second, this study did not compare the characteristics of inhibitory control across different game genres. Future research could further explore how FPS gaming experience influences various aspects of inhibitory control and whether these effects differ from those observed in other game types. Third, although the task was designed to approximate FPS-relevant response selection and inhibition demands under controlled conditions, it necessarily simplifies real gameplay. More immersive paradigms (e.g., VR-based environments) may further enhance ecological validity and clarify how visuomotor control processes operate in complex competitive settings.

## Conclusion

Motivated by the real-world demands of competitive FPS gameplay, where performers must rapidly select targets, coordinate eye-hand actions, and regulate responses under spatial uncertainty, FPS gaming experience was associated with faster response execution and more efficient gaze dynamics, whereas inhibitory accuracy was shaped primarily by spatial constraints (target distance and quadrant) rather than by a uniform group advantage. By linking a high-demand digital performance context to measurable component processes (spatial attention allocation, gaze orienting, and speed-accuracy regulation), our findings contribute to use-inspired basic research on how cognition operates in complex visuomotor environments. Future work leveraging finer-grained spatial sampling and longitudinal/training designs can further test the conditions under which experience-related differences generalize beyond FPS-relevant tasks and inform the development of ecologically valid assessments of visuomotor decision making.

## Data Availability

All raw data, materials, and analysis scripts are publicly available on the Open Science Framework at 10.17605/OSF.IO/RE5VM.
